# Supplementary material for: Dynamic recruitment of the curvature-sensitive protein ArhGAP44 to nanoscale membrane deformations limits exploratory filopodia initiation in neurons
Source: eLife. 2014 Dec 15;3:e03116. doi: 10.7554/eLife.03116 (PMC4381785; doi:10.7554/eLife.03116)
Supplement: Supplementary file 1. — Names and relative expression levels of 286 putative actin regulators. List depicting the expression levels of genes identified in the NCBI search in various tissues of the Human U133A/GNF1H Gene Atlas data set (gnf1h-gcrma unaveraged). Note that values are log-transformed. DOI: http://dx.doi.org/10.7554/eLife.03116.050 [file elife03116s001.docx]

Galic et al.; Supplementary File 1: Names, values and relative ratio of 89 neuronal enriched putative actin-regulators.

| **Gene ^a^** | **Adult**  **Brain** | **Spinal**  **Cord** | **Fetal**  **Brain** | **Adult Brain**  **Spinal Cord** | **Adult Brain**  **Fetal Brain** |
| --- | --- | --- | --- | --- | --- |
|  | (raw data) | (raw data) | (raw data) | (ratio) | (ratio) |
| RAN | 0.723116 | 0.211398 | 0.414608 | 3.248762773 | 2.034735677 |
| RAC1 | 0.476765 | 0.456062 | 0.349191 | 1.048824926 | 1.341448487 |
| RAB7A | 0.606755 | 0.389269 | 0.113251 | 1.65000781 | 3.115329589 |
| RAC1 | 0.255954 | -0.17109 | -0.104096 | 2.673277235 | 2.291131414 |
| MAPK3 | 0.396536 | 0.225357 | 0.008498 | 1.48312925 | 2.443644358 |
| HRAS | 0.529566 | 0.175174 | 0.282624 | 2.261476088 | 1.765801982 |
| MAP2K1 | 1.129592 | -0.157952 | 0.006715 | 19.38849058 | 13.27018569 |
| MAPK1 | 0.729343 | -0.069457 | 0.070298 | 6.292163513 | 4.560841713 |
| PTK2B | 0.447758 | -0.285962 | -0.28039 | 5.416515621 | 5.347465607 |
| RAP2A | 0.836357 | 0.0869 | 0.960962 | 5.616386671 | 0.750576564 |
| KRAS | 0.600098 | 0.03744 | 0.563563 | 3.653070049 | 1.087764798 |
| KRAS | 0.647304 | -0.787449 | 0.183174 | 27.21153243 | 2.911588531 |
| CDKN1B | 0.661742 | 0.384415 | 0.475745 | 1.893768986 | 1.534606382 |
| MINK1 | 0.427097 | 0.532902 | 0.290079 | 0.783781485 | 1.370938586 |
| PAFAH1B1 | 0.572115 | 0.501931 | 0.793962 | 1.175395436 | 0.600002417 |
| PAFAH1B1 | 0.836141 | 0.456679 | 1.015052 | 2.395863107 | 0.662352226 |
| TSC1 | 0.25627 | 0.120103 | 0.419812 | 1.368254862 | 0.686211513 |
| TIAM1 | 0.960582 | 0.639082 | 0.685384 | 2.09652478 | 1.884508063 |
| PKP4 | 0.409738 | 1.335463 | 0.167539 | 0.118651983 | 1.746622297 |
| PKP4 | 0.351037 | 1.525422 | 0.207518 | 0.066929102 | 1.391614675 |
| DAAM2 | 0.981199 | 1.552747 | -0.088844 | 0.268195817 | 11.75013889 |
| ARHGEF17 | 0.190408 | 0.223808 | 0.039497 | 0.925976575 | 1.415503671 |
| NF1 | 0.219061 | -0.096451 | 0.367557 | 2.067816517 | 0.710401713 |
| CIT | 0.639718 | -0.15206 | -0.053694 | 6.191245139 | 4.936418827 |
| PRKCZ | 1.099499 | 0.427978 | 0.816321 | 4.693761301 | 1.919455288 |
| SIPA1L1 | 1.013225 | 0.168452 | -0.186224 | 6.994762934 | 15.82883675 |
| KALRN | 1.514916 | -0.183919 | 0.436925 | 49.98445947 | 11.96715731 |
| ARHGEF4 | 0.981211 | 0.077375 | 0.060598 | 8.013753877 | 8.329386219 |
| WASF1 | 1.427674 | 0.305415 | 1.34822 | 13.25131568 | 1.200753884 |
| AGAP1 | 1.056474 | 0.006011 | 0.154521 | 11.23215273 | 7.979083318 |
| ARHGEF4 | 1.478097 | 1.04319 | 0.702296 | 2.72211833 | 5.967617791 |
| DNM3 | 1.231935 | 1.217609 | 1.360569 | 1.033536932 | 0.743645578 |
| TUBB4 | 1.67112 | 1.59483 | 0.899576 | 1.192037725 | 5.90940833 |
| AMPH | 1.681303 | 0.149139 | 0.916226 | 34.05367603 | 5.822064333 |
| DNM1 | 2.026499 | 0.708365 | 1.011258 | 20.80338469 | 10.3571675 |
| TUBB2B | 1.246856 | 1.371625 | 2.031104 | 0.750293182 | 0.164343299 |
| CDK5 | 1.308931 | 0.107444 | 0.324879 | 15.90329079 | 9.63944434 |
| SRGAP3 | 0.681014 | 0.047935 | 0.637505 | 4.296145684 | 1.105373376 |
| ABR | 1.214413 | 0.455517 | 0.767305 | 5.739789957 | 2.799677455 |
| ABR | 0.978024 | -0.103948 | 0.656652 | 12.07735967 | 2.095906961 |
| ARHGAP44 | 1.229123 | -0.123469 | -0.031406 | 22.52122451 | 18.21918728 |
| ARHGAP32 | 0.622086 | -0.160704 | -0.156976 | 6.064430175 | 6.012595673 |
| ARHGAP32 | 0.107033 | -0.047845 | -0.03245 | 1.428492617 | 1.378741983 |
| RND1 | 0.643191 | -0.034439 | -0.02781 | 4.760252612 | 4.688144616 |
| PKP4 | 0.835849 | 0.612633 | -0.067312 | 1.671921952 | 8.001308215 |
| PKP4 | 1.305463 | 0.118685 | -0.020695 | 15.37368577 | 21.19131953 |
| MAPRE3 | 1.115497 | -0.11453 | -0.001903 | 16.98349235 | 13.1038828 |
| MAPRE3 | 1.249096 | -0.108961 | -0.05564 | 22.8064138 | 20.17139807 |
| MYRIP | 0.536596 | -0.122772 | 0.391282 | 4.564235032 | 1.397378317 |
| CHRM3 | 0.690979 | -0.052798 | 0.420483 | 5.543409988 | 1.864215008 |
| TUBB3 | 1.156511 | 0.538548 | 1.450111 | 4.149186919 | 0.50862769 |
| TUBB3 | 1.145624 | 0.51417 | 1.318614 | 4.280100828 | 0.671444313 |
| TUBB2C | 0.874124 | 0.279099 | 1.037364 | 3.935727307 | 0.686688857 |
| TUBB2C | 0.704096 | 0.25659 | 0.921606 | 2.802244336 | 0.606024246 |
| TUBB2A | 1.61358 | 1.007965 | 1.71399 | 4.032877218 | 0.793578695 |
| ROCK2 | 0.449629 | -0.158746 | 0.308624 | 4.058588309 | 1.383582308 |
| GRLF1 | 0.146333 | -0.052914 | 0.235151 | 1.582147612 | 0.815045774 |
| FGD1 | 0.023898 | -0.00955 | 0.201421 | 1.080060293 | 0.664472482 |
| GNAI1 | 0.616387 | 0.76761 | 0.660557 | 0.70595497 | 0.903295819 |
| CDC42 | 0.644996 | -0.083585 | 1.683586 | 5.352799792 | 0.091497663 |
| CDC42 | 1.165875 | 0.242092 | 1.884304 | 8.390406459 | 0.191236594 |
| TNIK | 0.115409 | 0.077916 | 0.774928 | 1.090166921 | 0.219018601 |
| KIF3B | 0.012152 | -0.101714 | 0.632372 | 1.299768477 | 0.239761805 |
| NF1 | 0.029992 | 0.023313 | 0.151527 | 1.015497831 | 0.755901139 |
| BIN1 | 0.915098 | 0.538011 | 0.317803 | 2.382796755 | 3.956352691 |
| BIN1 | 0.833973 | 0.537365 | 0.299046 | 1.97973928 | 3.42710176 |
| BIN1 | 0.993508 | 0.689224 | 0.43911 | 2.015041524 | 3.584247574 |
| BIN1 | 0.832844 | 0.392981 | 0.183683 | 2.753360007 | 4.45821491 |
| BIN1 | 0.139961 | 0.082988 | 0.041972 | 1.140178901 | 1.253109435 |
| PAFAH1B1 | 0.037325 | -0.098401 | 0.269245 | 1.366866186 | 0.586246145 |
| TNIK | -0.011531 | -0.080016 | 0.080379 | 1.170806163 | 0.809263588 |
| PIK3R1 | 0.495728 | -0.156019 | 0.466538 | 4.484840476 | 1.069522684 |
| PIK3R1 | 0.497162 | -0.408705 | 0.779621 | 8.051318368 | 0.521844367 |
| PIK3R1 | 0.066482 | -0.110984 | 0.139586 | 1.50475571 | 0.845076451 |
| CTTN | 0.207032 | 0.492135 | 0.338667 | 0.518677012 | 0.738524658 |
| CDC42BPA | 0.021807 | 0.142211 | 0.250116 | 0.757872241 | 0.591140888 |
| ARHGEF12 | 0.318371 | -0.016844 | 0.595963 | 2.163789455 | 0.527725403 |
| GJA1 | 0.974261 | 1.569516 | 0.26518 | 0.253948119 | 5.117772779 |
| PTK2 | 0.352278 | 0.435878 | 0.028426 | 0.824897524 | 2.107909689 |
| CDC42BPB | 0.703728 | 0.618852 | 0.53998 | 1.215838804 | 1.457968027 |
| DOCK1 | -0.005986 | 0.537691 | -0.106715 | 0.285971662 | 1.261040399 |
| CDC42EP4 | 0.255015 | 0.368697 | 0.528703 | 0.769693821 | 0.532490667 |
| CDC42EP4 | 0.345684 | 0.444392 | 0.516663 | 0.796694832 | 0.674560645 |
| CDC42EP1 | 0.281419 | 0.352395 | -0.20512 | 0.849227404 | 3.065765975 |
| RHOB | 0.673105 | 0.870214 | 0.291112 | 0.635171495 | 2.409866586 |
| SLIT2 | 0.436732 | -0.212438 | 0.113622 | 4.4583073 | 2.104311361 |
| MRAS | 0.70937 | 0.206623 | 0.055577 | 3.182343095 | 4.506018807 |
| ARHGEF12 | 0.011715 | 0.004843 | 0.018269 | 1.015949217 | 0.985022158 |
| RAP2A | 7.53E-04 | 6.55E-04 | -0.004384 | 1.000225679 | 1.011898612 |
| HTT | 0.035328 | -0.019791 | 0.134662 | 1.13532186 | 0.795547289 |
| TNIK | -0.051098 | -0.028138 | -0.06767 | 0.94850582 | 1.038895823 |
| CTTN | -0.028282 | -0.011109 | -0.023692 | 0.961229299 | 0.989486789 |
| OPHN1 | -0.460318 | -0.121716 | 0.037252 | 0.458561934 | 0.318002109 |
| USP6 | 0.045348 | 0.156718 | 0.463381 | 0.773802271 | 0.38191525 |
| MAPRE3 | -0.288943 | 0.175626 | 0.016473 | 0.343108123 | 0.494975838 |
| PTK2 | -0.13071 | 0.129414 | 0.031024 | 0.549383991 | 0.689074217 |
| LMNA | -0.472595 | 0.204685 | 0.016995 | 0.210242252 | 0.323899293 |
| RIN1 | -0.164569 | 0.140269 | 0.013206 | 0.495635038 | 0.664087033 |
| MINK1 | 0.111468 | 0.177991 | -0.012219 | 0.857979675 | 1.329495893 |
| MINK1 | 0.022355 | 0.148092 | 0.013242 | 0.748622714 | 1.021205159 |
| INF2 | 0.105474 | 0.461962 | -0.022093 | 0.440060107 | 1.341426866 |
| FOXJ1 | -0.087435 | 0.150672 | -0.04396 | 0.577953636 | 0.904742517 |
| PIK3R2 | -0.02299 | 0.101038 | 0.487329 | 0.751574437 | 0.308802637 |
| CD82 | -0.13329 | 0.075637 | -0.021902 | 0.61812029 | 0.7737702 |
| S1PR1 | -0.093178 | 0.041704 | -0.035155 | 0.733023672 | 0.874937438 |
| PIP5K1A | 0.018661 | -0.005067 | -0.03608 | 1.056155828 | 1.134334132 |
| TIAM1 | 0.215354 | 0.023353 | 0.089853 | 1.555969214 | 1.335060664 |
| SRGAP2 | 0.106451 | 0.222903 | 0.777076 | 0.764800213 | 0.213488753 |
| GOLGA4 | -0.349865 | 0.241988 | -0.09305 | 0.255945206 | 0.553585875 |
| OCRL | 0.265358 | -0.078549 | 0.005067 | 2.207531961 | 1.820920562 |
| TWF1 | -0.153553 | 0.300949 | 0.352756 | 0.351154307 | 0.311667129 |
| PRKCI | -0.097426 | -0.114319 | 0.385461 | 1.039663985 | 0.328937207 |
| PRKCI | 0.252711 | -0.69133 | 0.372415 | 8.791055057 | 0.759094771 |
| RAPGEF3 | 0.054966 | 0.004903 | -0.032895 | 1.122181229 | 1.224224312 |
| ARHGAP6 | -0.163119 | -0.129142 | -0.148853 | 0.924747147 | 0.96768498 |
| GOLGA4 | -0.171195 | -0.21282 | -0.12953 | 1.100588575 | 0.908521062 |
| TNC | -0.002544 | 0.006855 | -0.007286 | 0.97859051 | 1.010978687 |
| ARHGEF11 | 0.032738 | -0.031905 | -0.029213 | 1.160494269 | 1.153323125 |
| RHOF | -0.045677 | -0.045 | -0.049393 | 0.998442364 | 1.008593117 |
| CDC42BPA | -0.043279 | -0.145276 | -0.0466 | 1.264727611 | 1.007676197 |
| EPHA5 | -0.017161 | -0.015733 | 0.442396 | 0.996717308 | 0.347090718 |
| SLC6A4 | -0.022722 | -0.03221 | -0.017157 | 1.022087319 | 0.987267862 |
| RAB11FIP3 | 0.168804 | -0.052731 | -0.028075 | 1.665463043 | 1.573544394 |
| PLCE1 | -0.189087 | 0.212517 | -0.050932 | 0.396639535 | 0.727520106 |
| PLCE1 | -0.226328 | -0.073009 | -0.167801 | 0.702556086 | 0.873922659 |
| CDC42BPA | -0.073853 | -0.015533 | -0.01489 | 0.874339301 | 0.873045745 |
| CDC42BPA | -0.022398 | -0.002685 | -0.026436 | 0.955623894 | 1.009341198 |

**^a^** Note that data is log-transformed and for some genes multiple probes are available. To calculate the ratio (eg. [adult brain] / [spinal cord]), the values need to be back-transformed (ie. raise 10 to the power of the number).
